# Addressing Disparities in Pre-Exposure Prophylaxis (PrEP) Access: Implementing a Community-Centered Mobile PrEP Program in South Florida

**DOI:** 10.21203/rs.3.rs-2799359/v1

**Published:** 2023-04-24

**Authors:** Stefani Butts, BreAnne Young, Jakisha Blackmon, Susanne Doblecki-Lewis

**Affiliations:** University of Miami, Miller School of Medicine; University of Miami, Miller School of Medicine; University of Miami, Miller School of Medicine; University of Miami, Miller School of Medicine

**Keywords:** HIV, Prevention, Implementation Science, Community-Based Participator Research

## Abstract

**Background:**

Pre-exposure prophylaxis (PrEP) for HIV prevention is highly effective, but disparities in PrEP access remain considerable, particularly among Black and Latino men who have sex with men (MSM). To address this, the University of Miami Mobile PrEP Program was created, offering mobile HIV prevention/PrEP services in areas throughout South Florida where HIV incidence is high and PrEP access is geographically limited. Using a community-centered participatory approach, the program strategized and executed expansion into the Liberty City neighborhood of Miami. This study qualitatively assessed factors affecting Mobile PrEP implementation as perceived by community stakeholders, clients, and program staff.

**Methods:**

Forty-one in-depth interviews were conducted with 21 Mobile PrEP clients, 10 key informants from local health organizations, and 10 program staff. Interview questions queried perceived organizational and positional barriers and facilitators to mobile clinic implementation. Service satisfaction, setting preferences, social factors, and likelihood of recommending Mobile PrEP were also assessed. A thematic content analysis was performed using the Consolidated Framework for Implementation Research (CFIR) taxonomy as the guiding constructs for the analysis.

**Results:**

Participant statements indicated that providing no-cost services, convenient location, program-covered rideshares, individualized patient navigation, and a community-centric approach to patient care, which included staff members with shared lived experiences to increase positive interactions and renewed trust among poorly served communities, were facilitators of PrEP access and intervention uptake. The importance of program familiarization with the community before implementation, particularly for Black and African American communities, who may experience unique barriers to accessing sexual healthcare was strongly emphasized by participants.

**Conclusions:**

The Mobile PrEP intervention was found to be an acceptable and accessible mode of HIV/STI preventive care. The importance of pre-implementation community engagement and preparation is emphasized. Future research is needed to refine understanding of the intervention’s components and evaluate implementation determinants in other highly impacted neighborhoods.

## Background

Pre-exposure prophylaxis (PrEP) for prevention of HIV is highly effective; daily adherence to PrEP medication can reduce the risk of HIV infection by more than 90 percent in real-world settings.(1, 2) Since the authorization of PrEP for HIV prevention by the Food and Drug Administration in 2012, PrEP utilization rates have increased consistently.(3) While increased PrEP uptake among highly impacted communities could substantially reduce the incidence of new HIV infections disparities in PrEP access relative to HIV diagnosis rates by geography, race and ethnicity are currently considerable.(3–6)

South Florida’s Miami-Dade and Broward Counties have among the highest incidence of new HIV infections, concentrated epidemics among Black and Latino MSM, as well as geographic/spatial concentration by zip code and census tract.(7) However, many clinic-based services remain geographically limited, have constrained hours, are logistically difficult to access, and are offered in facilities that may be perceived as stigmatizing.(8, 9) Further, recent immigrants, the uninsured, and people identifying as racial or ethnic minorities (groups overrepresented among new HIV cases) may have additional structural and social barriers to PrEP services.(10)

To address these barriers, we designed a mobile HIV prevention/PrEP service delivery system: the University of Miami Mobile PrEP Program (“Mobile PrEP”).(11) In 2020–2021, working with the Florida Department of Health and based on available HIV molecular cluster data, we planned and executed an expansion of Mobile PrEP services to a new site in the Liberty City neighborhood of Miami. Preparation for expansion to this new site included a community-centered participatory approach, working with stakeholders and community partners to develop an implementation strategy to address the key determinants influencing local PrEP engagement at this site. In this manuscript we describe the findings of our qualitative assessment of Mobile PrEP implementation determinants from the perspectives of community stakeholders, clients, and Mobile PrEP staff. These findings may serve as a guide for others contemplating delivery of PrEP services through mobile or other community-based approaches.

## Methods

### The Mobile PrEP Clinic

Established in 2018, the University of Miami Mobile PrEP Clinic is a customized mobile clinic that provides low-barrier PrEP, nPEP, STI, and HIV care services in five highly impacted neighborhoods in Miami-Dade. Services include assistance with transportation, general health screening and wellness, HIV and STD testing, laboratory monitoring and medication prescribing with patient-centered navigation, and ongoing support by multilingual staff.(10, 11) During the COVID-19 pandemic, the option for telehealth services for Mobile PrEP clients was also available. Initial sites for service delivery were chosen by evaluation of HIV incidence and prevalence by zip code as well as community consultation regarding areas with perceived low availability of HIV prevention services. In 2019, we began work with the Florida Department of Health to further refine selection of additional sites through use of HIV molecular cluster data.

### Molecular Cluster Prioritization

In collaboration with the Florida Department of Health (FDOH), Mobile PrEP was deployed as a cluster-response tool for geographic regions identified as priority locations for an ambulant clinic intervention based on the density of HIV cases within a given ZIP code or census tract area. Specifically, FDOH uses the Enhanced HIV/AIDS Reporting System (eHARS) to collect and maintain demographic and enhanced interview data from newly identified HIV cases. Additionally, FDOH uses the web-based bioinformatics tool Secure HIV-TRACE to compare genetic sequences of HIV and construct transmission cluster networks of persons with genetically similar strains of HIV, indicating recent and rapid transmission.(12) These sequence tests are analyzed for population-level trends in case-clustering and resistance to anti-retroviral therapies. Our preliminary assessment in 2019 found all active PrEP Mobile Clinic sites were in areas within the three highest density quintiles. From among the highest quintile areas without current Mobile PrEP deployment, novel priority location for Mobile PrEP was identified in the Liberty City area of Miami. Details of the derivation of the molecular cluster prioritization algorithm are described in a separate manuscript.

### Procedure

To gain a more comprehensive understanding of the determinants of PrEP delivery in the Liberty City neighborhood through the Mobile PrEP program, we conducted qualitative, in-depth, semi-structured interviews with key informants, Mobile PrEP clients, and clinic staff. Approval was obtained from the University of Miami Institutional Review Board. Forty-one interviews were conducted from July 2020 through March 2022: 21 Mobile PrEP clients; 10 key informants in leadership positions at local community-based health organizations; and 10 Mobile PrEP staff who were client-facing and/or in a managerial position with the program. Interviewees were approached in-person during normal clinic operations.

### Interview Guide

The interview guide was developed collaboratively by the study’s principal investigator and two junior researchers based on previous studies conducted by this group (Doblecki-Lewis et al, 2019). The guide was designed with the Consolidated Framework for Implementation Research (CFIR)(12) as a framework, and the Interview Guide Tool developed by Center for Clinical Management Research CFIR Research Team. Using a framework of 39 constructs over five domains, CFIR assesses determinants of implementation based on the context of the intervention(12). The guide included both open- and closed-ended questions to assess implementation factors including satisfaction and feedback regarding venue access and services received; preferences regarding setting to receive services; social factors impacting service initiation and maintenance (e.g., perceived stigma); and likelihood to recommend the service to others.

The informant interview guide queried perceived organizational and positional barriers and facilitators to mobile clinic implementation, including referrals to local clinical sites and impact on other clinical prevention services. Interview questions and stems for each group are presented in Appendix **A**.

All key informant and staff interviews were conducted through the teleconferencing software program, Zoom Video Communications using secured university accounts. Client interviews were completed using both virtual and in-person strategies to allow for the inclusion of clients with limited electronic access. Calls were conducted in English by two trained female staff members who each held a Master of Public Health (MPH) degree. Both staff members had upward of five years conducting and publishing qualitative research. Interviews were recorded and transcribed verbatim by the study team for qualitative analysis. Interview duration ranged from 30–60 minutes.

### Analysis

Thematic content analysis was performed using the taxonomy of the CFIR as the guiding constructs for the analysis. Two co-authors independently assessed the participant transcripts to identify and code key determinants. As with the interview guide, the codebook was based on a template developed by the Center for Clinical Management Research.(12) Transcripts were coded and content-analyzed line-by-line using the CFIR matrix; constructs and themes were identified within the CFIR domains. Two analysts coded and reviewed the transcripts in batches of five files at a time. Meetings were held after each batch for consensus coding, in which both coders reviewed the same transcript to ensure consistent code application, discuss coding disagreements, and review any newly identified codes. A third analyst was also asked to review the coded transcripts and assess the agreement and reliability of the identified themes after each round. An abridged version of the CFIR coding instrument was developed in the present study based on the feedback from participants, as some constructs did not apply to Mobile PrEP program implementation. All statements were coded into 33 of the 39 constructs.

The directionality and strength of the identified constructs were also assessed. Directionality was denoted using a positive, negative, or neutral valence to characterize the influence of the determinant on implementation. Strength was measured on a scale of zero to two to characterize the degree to which a determinant influences implementation. For example, a statement with a code of −2 would indicate a strong barrier to implementation. A consensus process was used to assign a rating to each construct based on the level of agreement among interview participants, the strength of language used, and use of concrete examples in their responses. A matrix of construct ratings was created once the findings from all participants were collected. The matrix was then used to identify potential patterns across participant groups, looking at the strength of the constructs characterizing Mobile PrEP, and the positive or negative influence they have on implementation.

## Results

A total of 41 individuals, including 10 community stakeholders, 10 Mobile PrEP staff members, and 21 Mobile PrEP clients participated in one-on-one interviews. Of the 39 CFIR constructs, 17 were identified as relevant barriers or facilitators to intervention implementation or PrEP utilization. As displayed in Table 1, responses related to barriers and facilitators varied significantly among stakeholders, staff, and clients, with stakeholders identifying significantly greater barriers to implementation when compared to clients, who largely identified facilitators to uptake in the community.

## Intervention Characteristics

### Relative Advantage

Participants in the stakeholder and client groups generally viewed Mobile PrEP as a useful method of service delivery within the community. Some features of the program, described by all participant groups as advantages, were convenience, hours of operation, ease of the process, and mobility: As one community key informant stated, *“…About this mobile thing, that’s the way! I mean, you can’t beat on the corner... [they] eliminate all the excuses that’s holding people back.”*

Several community stakeholders contrasted the Mobile PrEP system with negative experiences with attempting to get PrEP in other settings: *“It’s [acquiring PrEP] a difficult process at clinics… I mean the clinics that say they provide [PrEP]... I mean, I feel that they don’t provide it.”* A Mobile PrEP client also commented, *“The [Mobile PrEP] process goes just right. You don’t have a headache. You ain’t gotta worry about where your medicine[‘s] at. Just come get your checkups and then you’re good to go...”*

Staff echoed this perception: “*The feedback that we get is that it [Mobile PrEP] does overcome many of the barriers that are encountered in other systems… it does enable people to get on PrEP who might otherwise not be able to... it does meet a need in the community for a niche for services that are available this way.”*

### Adaptability

Ability to provide services in varied settings, including clinic-based, mobile, and virtual platforms, were cited as important factors for engagement and accessible care. Most participants found the mobile clinic’s process to be flexible and tailorable to clients’ needs. A client noted, *“… This can be a big help… promoting telehealth. Because right now you do the video on your cell phone. You still got to come in and do the labs… But, then after that, they can just send the prescription to whatever pharmacy you want…”*

Staff also reinforced the adaptability of the program. One staff member commented, *“It’s super easy for most patients… we can provide them with a Lyft to come in. If they can’t come in, we have telehealth services… We can send them a box to get their labs done… Whatever barrier you might have, we try to find a way to overcome that…”*

Some community stakeholders suggested that it is necessary for program/staff to adapt to the level of client knowledge and understanding seen in this specific community to encourage uptake of the Mobile PrEP intervention. These participants believed that knowledge of PrEP as HIV prevention was low among their community and that this may create a barrier to utilization of Mobile PrEP services. As one participant stated *“...You got to meet them where they’re at. You have to go reach the population where they are. You know they don’t know anything about PrEP. You have to start from scratch.”*

### Complexity

While many stakeholders understood that PrEP delivery is a complex process involving clinical evaluation, laboratory studies, and medication prescription, they perceived that the Mobile PrEP strategy alleviated much of this complexity from the client perspective. As one community stakeholder indicated, *“I think that accessing PrEP is cumbersome… There’s a lot of requirements that people need to meet in order to get it …and logistic steps that they need to take…”*

However, of clients interviewed, 20/21 participants reported that acquiring PrEP through the Mobile PrEP program was *“easy,”* and many clients attributed this to ancillary services provided by the program, such as navigating insurance and prescription routing to and coordination with pharmacies to fill their prescriptions. One PrEP client indicated, *“It was extremely easy…when I went to my next appointment, they had [my medication] on hand for me. That was great!”*

Staff participants echoed responses from the client group. They reiterated that those processes associated with completing a PrEP visit and obtaining medication were designed for a convenient and uncomplicated client experience by reducing client burden as much as possible. One staff member commented, *“it’s very easy to access our services, we’re very accommodating, we’re able to squeeze them in… same week or same day sometimes. So, I think it’s very easy for patients...”*

Staff relayed, however, that creating and maintaining this low-barrier environment for clients could be challenging. As one staff member stated, *“…It might seem seamless but, it’s not seamless… there’s a lot of work that goes into it,”* and another commented, *“Mobile PrEP is fairly complicated. It’s a pretty extensive operation. There are a lot of moving pieces, a lot of different people that go into the process of getting the patient their PrEP. So, it does come with its challenges…” S*ome participants in the staff group believed that certain aspects of these protocols could be simplified: *“I think there is room for streamlining and steadiness… it makes it hard… when you have to adjust to the situation and change your protocols.”*

### Design Quality & Packaging

Participants in the key informant and client groups believed that a mobile unit situated in the community, in and of itself, was attention grabbing. These participants believed that a mobile unit may promote curiosity among community members which, in turn, may lead to engagement with the intervention and eventually utilization. As one community stakeholder stated, *“It’s a good idea. I feel like if a person needs PrEP, the PrEP mobile will be something that will get their attention. It’s a good way of getting people to see what PrEP is about…”* Indeed, another Mobile PrEP client noted that they accessed services because of Mobile PrEP’s visibility in the community, *“Yeah, I noticed it [the mobile unit] every time I passed by…”*

Clients suggested that confidentiality and having positive interactions with Mobile PrEP staff were important factors that may influence utilization. Some clients described initial hesitancy to seek services with Mobile PrEP. However, it was stated that these feelings dissipated after engaging with staff and familiarization with the mobile clinic. Some aspects of the mobile clinic design, such as ability to enter and exit through different doors, were noted as positive factors *“… it made me more comfortable when I seen that… you enter through one side and use the exit through the back… and then the guys did their job pretty nice.”* Space constraints, a feature inherent to a mobile clinic, was viewed as a potential drawback to the intervention. One staff member relayed, *“People have expressed that they don’t like the mobile unit because of the space itself, it’s very small. I guess out of claustrophobia or the issue of confidentiality…”*

### Cost

Perceived cost of PrEP services was a reported barrier for many clients prior to communicating with Mobile PrEP staff. One client expressed *“If it’s not close I ain’t going and if I got to pay I ain’t going.”* The ability to access care through the Mobile PrEP program at no cost was expressed as an advantage by many clients, stakeholders, and staff. One client recommended, *“Always tell us it’s free. When she [Mobile PrEP CHW] was coming door-to-door, she let you know the service is free. Just come on out…” A Mobile PrEP staff member reinforced, “An advantage is that we are completely free, whether you have insurance or not the patient doesn’t have to pay anything.”*

## Outer Setting

### Needs & Resources of Those Served by Mobile PrEP

Statements made by key informant and client participants highlighted their knowledge of the HIV prevalence in the area and awareness of the impact of HIV in their community. Also, participants noted that there was a dearth of initiatives focused on HIV prevention and providing PrEP. They believed that there was a need for Mobile PrEP and that these factors would drive its utilization. As one client commented *“…it’s [HIV] more prevalent in our community than most. People are sexually active at an earlier age […] just the availability to have a site that people can go into [and] doesn’t take long…”* A community stakeholder reinforced, *“…our HIV rates in Miami are really, really, high compared to other places. So, yes, we do need to prevent… because people are unaware of their status... just having PrEP as a method or having people out there testing people for HIV…”*

Participants in the staff group generally believed that Mobile PrEP meets the needs of the communities they serve. Many participants reported that experiential and cultural similarities between Mobile PrEP staff and community members made Mobile PrEP a good fit for its clients. They believed that clients identify with staff because of shared experiences and that Mobile PrEP was a needed and valuable resource. This belief was informed by client feedback and available data. As one staff member commented, *“A lot of patients have their own lived experiences, they come with very traumatizing stories and the way that we address those patients that come with those circumstances is very unique to our program. Not every program has people that have the lived experiences of their patients and that can lend that extra support… I believe that that is what keeps a lot of our patients wanting to come back to us… they know they have a trustworthy person that they can be completely honest with in regards to the sexual health and the things they may have done and regrets they may have… they know that we won’t judge them, and we’ll get them help as best as we can.”*

Another staff member indicated, *“Many of us are part of the community or have experienced getting healthcare in Miami and know the barriers that exist. We make it easy for the patient.”* Another reinforced the importance of having staff with language fluency appropriate for the community, *“We have different patient populations. We have Spanish-speaking populations. We have some Creole-[speaking] patients… and you know we meet that need through our staffing. Because we have a very diverse population in South Florida…”*

### External Policies & Incentives

Some key informant and staff participants commented on how external policies align with the Mobile PrEP intervention and how they may influence uptake. Key informant participants believed that ancillary policies that function to increase HIV testing would translate into uptake of the intervention. Participants from the staff group believed Mobile PrEP was in direct alignment with the national “Ending the HIV Epidemic” initiative as well as the Florida Department of Health’s “Four Pillars” initiative, and that this facilitated its implementation, *“The Ending the HIV Epidemic initiative has been very helpful because its goals are very much in alignment with the goals of our program… thinking about the metrics for elimination of HIV transmission, that gives us a concrete framework in which to place our intervention and to say that we are also aligned with those… and I think it’s helpful to put that context around the intervention as well. We’re also in alignment with the four pillars of the Florida Department of Health in terms of HIV prevention and I think it’s important to present our information and our program in that way… It helps to provide a way to see its wider application.”*

Staff also noted that Mobile PrEP addressed gaps in HIV prevention services that were recognized by outside agencies, *“It was the response that everyone was asking for, to get to these communities that weren’t able to access healthcare for other reasons. So, I think that we are able to do that… to go to these areas that have the high incidences of HIV.”*

## Inner Setting

### Structural Characteristics

Participants from all groups reported that transportation was a significant barrier to accessing health care. As one community informant stated, *“There [are] limited public transportation options, which makes it more difficult to get to the clinic. On top of that, you need to have time to prepare to get there.”* Participants indicated that the mobile nature of the intervention, in addition to no-cost transportation services offered, mitigated this barrier. Further, statements made by participants from the client group suggested that clients were more comfortable utilizing Mobile PrEP as it was located in familiar and convenient surroundings. One client stated, *“If it be any other way I’d have to travel. I’d have to get on a bus, or two buses to get an HIV test… when the services are provided here in the neighborhood, in the community, it works out very well.”*

Another client echoed, *“…Because it’s close to home, I’m still in my neighborhood and I feel safe.”* Staff agreed that transportation could be a barrier for many potential clients, and that the Mobile PrEP program was able to overcome this barrier by positioning services in the neighborhood. As one staff member noted, *“…We go out into the community. We go out to the areas that are most affected. Some patients… don’t have the resources to go to medical settings like the health department.”*

### Networks & Communications

Many participants, across groups, recognized the importance of forming collaborations with organizations that provided additional services and who were already established within the community to allow for expanded bundling and fostering trust, as one participant stated, *“Collaborate with someone that’s present in the community... Because we can’t do this ourselves. We have to learn how to collaborate.”* Some participants believed that collaborating with local drug treatment centers, specifically, may be a high yield strategy to encourage uptake of Mobile PrEP services. It was stated that treatment centers were uniquely positioned to deliver health education to their clients and so collaborations with these organizations may prove valuable. As one stakeholder iterated: *“You can go to a drug treatment center that’s not providing PrEP… you get that MOA with the center… the treatment center will let them [their clients] know… th’y’ll put it on the schedule that PrEP mobile will be here on October* ^*26*^*th from nine to five. They would then be charged to teach what PrEP is and then allow the clients to make a decision whether th’t’s something they want.”*

Participants also recognized that collaborating with established organizations assists the program with identifying community-specific gaps in care. As one staff participant mentioned, *“…collaborating with our public health programs helps us to know where those [high incidence] spots are… where we’re going to meet that target population.”* Staff also noted the influence of community municipal support on the program’s development: *“The City of Miami Beach helped us launch the PrEP program… they were involved in helping us launch our PrEP program. They saw the need for it in their city…”*

### Culture

Participant statements highlighted how the history and culture of the community may influence intervention uptake. Participants suggested that past experiences with the medical community and generational trauma, especially among communities of color, may act as a barrier to engagement with the healthcare system. As one participant stated*“ “...Especially in communities that are predominantly African American, the’e’s a …history of mistrust towards health care...”*

Other participants discussed the influence of cultural norms surrounding sex and sexual identity, and how the associated stigma can deter many from utilizing preventive measures and services offered by mobile PrEP. One participant noted, *“… and then the stigma with the words HIV or AIDS... ‘Cause I get people when I tell them about PrEP... they’re like “Oh no, not no HIV … I’m not gonna come in contact with somebody like that.”*

Again, although participant statements did not indicate there was stigma related to utilizing the mobile PrEP intervention specifically, stigma associated with lack of knowledge and understanding of HIV prevention (PrEP) versus treatment (ART) was also believed to inhibit uptake of the intervention. As one client participant relayed, *“Some people are afraid to take the medicine ‘cause they don’t want nobody to know they’re taking it. ‘Cause people might think they have HIV. Especially family members [...]”*

## Implementation Climate

### Tension for Change

There was a consensus among all groups that there is an urgent need for HIV prevention initiatives in this specific community and South Florida generally. Further, participants believed available interventions needed increased promotion and visibility. Statements made by participants conveyed exasperation at current HIV rates and reluctance of the community to utilize preventive measures. As one participant stated, *“Everybody needs to be aware of it. You mentioned at the beginning that the numbers done went up. Y’all need to get on this van, I don’t care who watching! … and people are going to be hesitant, but we have to stay proactive… We got a clinic here, we need to come, people are sick… y’all got to come get the help!” Others expressed urgency for service roll-out, “I feel like it’s great for the areas that they are placing it in, but I think they should expand, expand, expand…”*

While staff and community stakeholders clearly expressed a recognized community-level HIV risk and need for new PrEP service options, several participants noted that potential clients did not always recognize individual risk and did not feel urgency to utilize HIV prevention strategies, *“…Even with condoms… I have people saying, ‘I don’t need any condoms.’ I’ll say, ‘Are you sexually active?’ ‘Yes.’ … ‘Do you know their status?’ ‘No.’ So, I say ‘You need condoms! Do you get tested often?’ ‘Maybe,’ …You know that kind of thing. So, the ignorant part of why they should have this extra protection is still there”*

### Compatibility

Key informant participants believed strongly that client-facing staff needed to be compatible with their community. They recommended that program staff familiarize themselves with the Liberty City community prior to program initiation. Many felt that it would be optimal to utilize community health workers for initial community engagement and to guide the program’s community relations. Some suggested that available initiatives did not make enough of an impact because persons implementing these initiatives lack an understanding of the community. As one community key informant noted: *“The programs that they have in place, do not relate to the population that they are serving. They have no connection with the population that they’re serving.”* Another advised, *“...Make sure that your outreach workers [have] a nonjudgmental attitude because [they] might walk up to somebody that will cuss them out. They got to be able to keep their cool.”*

All participants from the client group, who had interacted with and received services from Mobile PrEP, recalled positive interactions with Mobile PrEP staff. These participants expressed satisfaction with services they received and felt that the model of care was appropriate for them. There was consensus that Mobile PrEP was compatible with their community. One client participant stated, *“I don’t have any complaints about any of you guys… You educate us on any new services and anything that is provided for us here [in] low-income neighborhoods… The staff is always on point.”* Other clients mentioned trust: *“I trust you guys. I didn’t have to worry…”* and a positive approach, *“…It was chilling, you know what I mean. They wasn’t all negative you know what I mean…”*

## Characteristics Of The Individual

### Knowledge & Beliefs about the Mobile PrEP Intervention and PrEP Medication

Individual attitudes towards the mobile PrEP intervention and participant awareness of Mobile PrEP and its benefits varied among interview groups. Statements made by client participants indicated that this group had little to no knowledge of mobile PrEP or its services prior to placement of the mobile unit in the area. However, after engaging with the intervention, client participants seemed to develop a positive view of mobile PrEP. Most client participants reported increased knowledge and understanding of mobile PrEP services after interacting with mobile PrEP staff. As one client described: “... They *[mobile PrEP staff] made it easy for me to understand what it was that they were doing in the community… And it was pretty cool. It was a nice experience overall.”* Other client participants described staff’s approach to education and offering mobile PrEP services improved their perception of the intervention and encouraged uptake: *“To be honest, at first I was a little unsure but after dealing with the workers there, I was 100% confident. It was nothing else to think of after that.”* Additionally, clients seemed to recognize and appreciate efforts made by staff to talk to and not at them. As another client noted: *“Everybody was nice, they gave me information …and they gave me the option to go ahead and explore PrEP.”*

Key stakeholder and staff participants described how knowledge and beliefs about the PrEP medication itself, rather than the mobile PrEP intervention, may influence community utilization. For example, although staff members were aware of the strength of evidence in favor of PrEP and its efficacy, and were in support of the mobile PrEP intervention, they believed that lack of knowledge about PrEP among community member might hinder uptake: *“...I’m not necessarily sure if PrEP education in every community is the same. I know in White MSM communities and even White Latin MSM community, people are very educated on PrEP and know that they need it. But in other communities this is something completely new to them.”* Stakeholders described similar concerns: *“From my experience… just from what I’ve done as far as outreach… the average African American male or female know nothing about PrEP.”*

### Identification with the Organization

Program staff were a significant positive influence in program implementation among participating clients. Many clients expressed that their positive experiences with the staff fostered commitment and adherence to the intervention, with one client stating, *“They made me feel like I wanted to get those services. They made me want to […] keep coming back.”*

When asked about their willingness to endorse the mobile PrEP clinic, stakeholders remarked that the intervention should demonstrate reliability and consistency before they would align themselves with the initiative. Many described past experiences where clients, who were referred to other organizations, faced barriers to care when they attempted to access PrEP. *“For me, with referring clients anywhere [I think] is this a trustworthy and credible establishment?” and it doesn’t mean that you have to be like the most known organization in the world or the community, but like the credibility…”*

## Process

### Planning & Engaging

Clients felt that community engagement should be conducted consistently and believed that community familiarization would encourage uptake of the initiative by community members: *“In the future, what you can do is broadcast mobile PrEP more often and let them [the community] know they can go through UM.”*

Similarly, there was general consensus among stakeholders that community engagement should be made a top priority, with many remarking that engaging the community would foster trust in the PrEP program, and would be a critical first step to community buy-in: *“Definitely knowing the community. Wherever you’re going to be you have to know the people in the community… instead of going there and wanting to provide the service right away, I think if there would be an opportunity first for them to get to know who’s there… So, doing that footwork and walking around and seeing who’s here… what kind of people live here.. and things like that.”*

### Executing & Reflecting

Many clients recognized that they themselves could function to promote the intervention and asserted that they would and did promote mobile PrEP: *“...a lot of those cats that was coming through… Cause they saw me going over there and was asking me… I would tell them what was going on and they went and got checked out. Just because they saw me over there. I was like yeah man I think you should. Everybody was like, “Hey, if Skeet went over there… Skeet a cool dude. Man let’s go over there and check it out”*

When asked, all interviewees asserted that they would recommend the PrEP program to members of their community and social networks: *“As far as me getting treated with respect… I have no other choice but to recommend [mobile PrEP] to people’’.*

## Discussion

Our findings from this in-depth exploration of the experiences of Mobile PrEP clients, staff, and key community stakeholders illuminate a range of barriers and facilitators that may influence implementation of a mobile clinic for HIV prevention services within one resource poor and medically underserved community in South Florida. Framed by the CFIR, participants’ narratives detailed how aspects of the intervention’s design and community characteristics work to overcome or preserve structural and social barriers to intervention access. Understanding these determinants is critical for the design and successful implementation of future HIV/STI prevention initiatives within similar settings. Additionally, participants have offered several recommendations for improving intervention uptake within this community.

Affordability is an already well characterized individual-level barrier to PrEP uptake(13) which the Mobile PrEP intervention was designed to overcome. Indeed, low/no cost services provided by Mobile PrEP was described, by all three participant groups, as a major facilitator of access and uptake. However, participants from the client group believed that community members who have not interacted with the intervention may perceive high costs for Mobile PrEP services which may inhibit buy-in and engagement. These views are supported by the literature.(14–17) A narrative review that assessed barriers to the wider use of PrEP in the US found that financial concerns, held by individuals before they attempted to access PrEP specifically, was a common barrier to PrEP seeking reported by most studies included in the review. (18) These findings indicate that awareness of low/no cost services may be a requisite precursor to intervention utilization and directly linked to intervention uptake. Future prevention initiatives that will provide free services within a similar context, should aim to disseminate feature-specific information (i.e., that services are provided at no cost) among all community members and not just those suitable for intervention use. This may help to build both community knowledge of and community-level support for such initiatives.

Positioning Mobile PrEP in a location that was convenient and easy for members of the community to access and offering program-covered rideshares were also viewed as major facilitators of intervention uptake by participants from all three groups. There was a clear preference among client participants for accessing care in their neighborhood. Further, client and stakeholder participants iterated that vehicle ownership and accessibility was low in the community and public transportation in the Miami metro area was unreliable and cumbersome to use. Studies of healthcare access, including those assessing access to PrEP among priority and low-income populations, have found lack of transportation to be a significant barrier.(18–21) Further, Miami-Dade’s public transit system lags behind those of many other US cities in accessibility and convenience.(22, 23) These findings suggest that overcoming geographic barriers, either by positioning of services (as in Mobile PrEP) or provision of transportation(24), is critical to facilitating access to care for priority populations.

Policy barriers related to insurance and pharmacy benefits can prevent the receipt of PrEP medication after receipt of a PrEP prescription.(20, 25) A study of new PrEP prescription holders in New York City reported that systemic issues, including failed attempts at navigating insurance/pharmacy systems, accounted for the largest category of reasons for discontinuation.(25) This is congruent with our finding that individualized patient navigation, aimed at overcoming insurance/pharmacy related barriers, was an important component of the Mobile PrEP intervention. Participants from all three groups in our study described Mobile PrEP as uncomplicated and easy for clients to use. Factors that influenced this view were Mobile PrEP’s ancillary services, such as navigating client insurance, identifying financial assistance programs, and connecting with pharmacies to coordinate prescription routing. This was in contrast to experiences reported by some client and stakeholder participants who had encountered insurance/pharmacy barriers that stopped them from receiving medication in other settings. Patient-specific tailoring of services, including appointment times at non-traditional hours and flexible meeting modalities (e.g virtual appointments) were also viewed as highly acceptable characteristics that reduce common barriers to engaging in care. Future research is needed to further assess the contributions of these components, to increasing intervention uptake.

Participants in the key informant and client groups relayed that the appearance and presence of the mobile unit in the community, in and of itself, was attention grabbing. They believed that it stimulated curiosity among community members which may facilitate individuals’ engagement with and introduction to the Mobile PrEP intervention. Other studies have provided some description of how the appearance of mobile clinics, in related contexts, may affect client acceptance of an intervention.(26) However, the present study provides a better understanding of how mobile clinic appearance may increase community engagement. While its’ outward appearance was found to be a strength among participants, there were also discussions of how its design may pose unique challenges to maintaining client confidentiality. Participants in the client group suggested there was increased potential to overhear private conversations within the finite design of the mobile clinic, which may act as a deterrent to intervention uptake. Reviews of similar mobile health clinic interventions have reported such spatial and structural constraints, which are inherent to operating in a confined space.(27) Considerations for obscuring noise and scheduling, however, can mitigate many of these barriers. As examples, playing ambient sound and staggering appointment times, to mask conversation and minimize client occupancy, are strategies that can be employed to protect client privacy in this and similar nontraditional settings.

Emphasis was placed on the importance of community familiarization, prior to intervention implementation, from stakeholder participants. For key minority populations, such as Black and African American communities, experiences of stigma, systemic bias, and fears of rejection or ostracization can be crucial barriers to seeking out PrEP and thus a barrier to intervention uptake. Participant statements highlighted how Black culture and the history of the Black experience in America may negatively influence intervention uptake. Mistrust of the healthcare system stems from a long history of medical mistreatment among Black Americans that dates back to the era of slavery and persists to this day.(28) Recent studies show Black patients are consistently undertreated for pain, receive less time with providers, and experience more structural barriers to care relative to patients of other racial demographics.(29) Community-led services, such as Mobile PrEP, are a potential solution that has been shown to be feasible, popular, and effective at overcoming systemic barriers to achieve improved health outcomes.

Notably, as fellow members of the communities served by Mobile PrEP, many participating staff members believed their shared lived experiences in accessing sexual healthcare within the local context provided them with the insight needed to deliver care to a traditionally hard-to-reach patient population. This community-centric approach to patient care has facilitated greater positive interactions between clients and staff and fostered renewed trust among clients who have been poorly served by the health care system. Staff also relayed, however, that creating and maintaining this low-barrier environment for clients could be challenging. Further public health efforts, such as nesting PrEP education within broader prevention and health promotion efforts, will be needed to simplify and streamline the process of community-based PrEP initiation.(30, 31) Ultimately, these reports highlight the need for sociocentric familiarization and suggests academic knowledge alone should not be the sole foundation on which community initiatives are developed.

This study’s strengths include its exploration of a novel model for PrEP and sexual health care delivery within a community disproportionately impacted by HIV and a wide breadth of stakeholder perspectives to understand factors that impact implementation and uptake. This study purposefully evaluated implementation within one community, in anticipation of a planned expansion of services to that neighborhood. While some of the domains described may apply to mobile PrEP implementation in many communities, other findings are likely community specific. For this reason, we believe that assessment and preparation of each community is an essential component of mobile service implementation. Additionally, not all community stakeholders interviewed were PrEP candidates and, while invested in enhancing community health outcomes, may have different perspectives than the intended users of the service.

Overall, participants in our study found the Mobile PrEP intervention to be an acceptable and accessible mode of HIV/STI preventive care for this community. Work to understand determinants and priorities in a neighborhood prior to expansion of services provided useful information for implementation. Future research to refine understanding of the contributions of different components of the intervention and to evaluate implementation determinants in other neighborhoods highly impacted by HIV are needed.

## Figures and Tables

**Table 1:** CFIR Constructs & Ratings† Identified by Participants in the Mobile PrEP Implementation Interviews

	Clients	Stakeholders	MP* Staff
**INTERVENTION CHARACTERISTICS**			
**Relative Advantage**	**+ 1**	**+ 1**	**+ 2**
**Adaptability**	**+ 1**	**+/−**	**+ 1**
**Complexity**	**+ 1**	**−1**	**+/−**
**Design Quality & Packaging**	**+ 1**	**+ 1**	**+ 1**
**Cost**	**+ 1**	**+ 1**	**+/−**
**OUTER SETTING**			
**Needs & Resources Served by Org.**	**+ 1**	**−1**	**+1**
**External Policy & Incentives**		**−1**	**+/−**
**INNER SETTING**			
**Structural Characteristics**	**−1**	**−1**	**+/−**
**Networks & Communications**	**+ 1**	**+ 1**	**+1**
**Culture**	**−1**	**−1**	**+1**
Implementation Climate			
**Tension for Change**	**+ 1**	**−1**	**+ 1**
**Compatibility**	**+ 1**	**+/−**	**+ 2**
**CHARACTERISTICS OF THE INDIVIDUAL**			
**Knowledge & Beliefs about the Intervention**	**+/−**	**−1**	**+/−**
**Individual Identification with Organization**	**+ 2**	**−1**	**+1**
**PROCESS**			
**Planning & Engaging**	**−1**	**+ 1**	**−1**
**Executing & Reflecting**	**+ 1**		**−1**

† Green and + indicate facilitators/positive progress in implementation process

Red and − indicate barriers/incomplete progress in implementation process

+/− indicates a near equal mix of positive and negative comments

* Mobile PrEP abbreviation

## Abbreviations

PrEP: Pre-exposure prophylaxis
nPEP: non-Occupational post exposure prophylaxis
HIV: Human immunodeficiency virus
STD: Sexually transmitted disease
MSM: Men who have sex with men
FDOH: Florida Department of Health
eHARS: Enhanced HIV/AIDS reporting system
CFIR: Consolidated Framework for Implementation Research
